# Metabolic Dysregulation and Neurovascular Dysfunction in Diabetic Retinopathy

**DOI:** 10.3390/antiox9121244

**Published:** 2020-12-08

**Authors:** Thangal Yumnamcha, Michael Guerra, Lalit Pukhrambam Singh, Ahmed S. Ibrahim

**Affiliations:** 1 Department of Ophthalmology, Visual and Anatomical Sciences, School of Medicine, Wayne State University, Detroit, MI 48201, USA; michael.guerra2@med.wayne.edu (M.G.); plsingh@med.wayne.edu (L.P.S.); 2 Department of Pharmacology, School of Medicine, Wayne State University, Detroit, MI 48201, USA; 3 Department of Biochemistry, Faculty of Pharmacy, Mansoura University, Mansoura 35516, Egypt

**Keywords:** hyperglycemia, diabetic retinopathy, neurovascular dysfunction, metabolic deregulation, glycolytic pathway, endothelial cell, glycolytic overload

## Abstract

Diabetic retinopathy is a major cause of ocular complications in patients with type 1 and type 2 diabetes in developed countries. Due to the continued increase in the number of people with obesity and diabetes in the United States of America and globally, the incidence of diabetic retinopathy is expected to increase significantly in the coming years. Diabetic retinopathy is widely accepted as a combination of neurodegenerative and microvascular changes; however, which change occurs first is not yet understood. Although the pathogenesis of diabetic retinopathy is very complex, regulated by numerous signaling pathways and cellular processes, maintaining glucose homeostasis is still an essential component for normal physiological functioning of retinal cells. The maintenance of glucose homeostasis is finely regulated by coordinated interplay between glycolysis, Krebs cycle, and oxidative phosphorylation. Glycolysis is the most conserved metabolic pathway in biology and is tightly regulated to maintain a steady-state concentration of glycolytic intermediates; this regulation is called scheduled or regulated glycolysis. However, an abnormal increase in glycolytic flux generates large amounts of intermediate metabolites that can be shunted into different damaging pathways including the polyol pathway, hexosamine pathway, diacylglycerol-dependent activation of the protein kinase C pathway, and Amadori/advanced glycation end products (AGEs) pathway. In addition, disrupting the balance between glycolysis and oxidative phosphorylation leads to other biochemical and molecular changes observed in diabetic retinopathy including endoplasmic reticulum-mitochondria miscommunication and mitophagy dysregulation. This review will focus on how dysregulation of glycolysis contributes to diabetic retinopathy.

## 1. Introduction

Diabetic retinopathy (DR) is a specific neuroretinal and microvascular complication of both type 1 and type 2 diabetes, the prevalence of which is strongly related to both the duration of diabetes and the level of glycemic control [1,2]. According to World Health Organization (WHO), DR accounts for an estimated 15–17% of total blindness in Europe and the USA [3], with no apparent ethnic variations in the occurrence of vision loss [4]. As the number of cases of diabetes is expected to increase from 366 million in 2011 to 552 million in 2030 [5], DR will become an even larger problem in the near future [6]. In the modified Airlie House classification of DR, human DR begins from non-proliferative DR (NPDR), characterized by increased occlusion, capillary dropout, vascular permeability, and number of microaneurysms (swellings on the side of tiny blood vessels that wax and wane). With increasing severity, DR progresses to the advanced stage of proliferative diabetic retinopathy (PDR), characterized by the formation of a fibrovascular membrane (FVM) and aberrant neovascularization on the surface of the retina rather than physiological vascularization within the retina itself [7]. At this stage, patients are at risk of blindness due to relentless abnormal fibrovascular proliferation with subsequent bleeding and tractional retinal detachment. The Diabetic Retinopathy Study (DRS) recommended prompt pan-retinal photocoagulation (PRP, also called scatter laser treatment) in all patients with PDR who meet the high-risk criteria (HRC) of having neovascularization that is at least one-half disc area in size associated with pre-retinal or vitreous hemorrhage [8]. However, PRP causes decreased visual acuity, reduced dark adaptation, and worsening of diabetic macular edema (DME) over the course of 2 years [9]. The advent of anti-vascular endothelial factor (VEGF) agents, according to The Diabetic Retinopathy Clinical Research Network (DRCR.net) studies, has added a potentially viable alternative or adjunct tool to PRP in the armamentarium of treating PDR and DME, through at least 2 years of treatment [10,11]. However, a transition from angiogenesis to fibrosis can occur when the vitreous level of VEGF decreases and the levels of connective tissue growth factors (CTGF, CCN2) increase [12]. In addition, subsequent prospective studies showed that the resistance to anti-VEGF has become a major clinical concern in patients receiving long-term therapy [13,14,15,16,17]. This is in part due to the upregulation of alternative pro-angiogenic factors to overcome VEGF blockade [18,19]. Hence, new strategies need to be developed with the goal of helping patients for whom anti-VEGF treatments are not effective.

The pathogenesis of DR is regulated by numerous factors via integrated molecular signaling pathways and cellular processes [20]. However, disruption of glucose homeostasis, an essential component for normal physiological functioning of retinal cells [21], plays one of the most critical roles in the development of DR. The maintenance of glucose homeostasis is finely regulated by coordinated interplay between glycolysis, Krebs cycle, and oxidative phosphorylation (OxPhos). Dysregulation of glucose homeostasis in many retinal cell types has been linked to the neural and microvascular complications of DR [22]. Glycolysis is the most conserved metabolic pathway in biology that is used by all prokaryotic and eukaryotic cells in generating energy from glucose [23]. This metabolic pathway is tightly regulated to maintain a steady-state concentration of glycolytic intermediates; this regulation is called scheduled or regulated glycolysis. However, an abnormal increase in glycolytic flux generates large amounts of intermediate metabolites that can be shunted into different damaging pathways including polyol pathway, hexosamine pathway, diacylglycerol-dependent activation of the protein kinase C pathway, and advanced glycation end products (AGEs) pathway. In addition, disrupting the balance between glycolysis and oxidative phosphorylation leads to other biochemical and molecular changes observed in DR, including endoplasmic reticulum–mitochondria miscommunication and mitophagy dysregulation. The following sections will discuss the contribution of glycolysis dysregulation to these pathways in DR.

### 1.1. General Structure and Function of Neurovascular Unit of Retina

**Figure 1:**
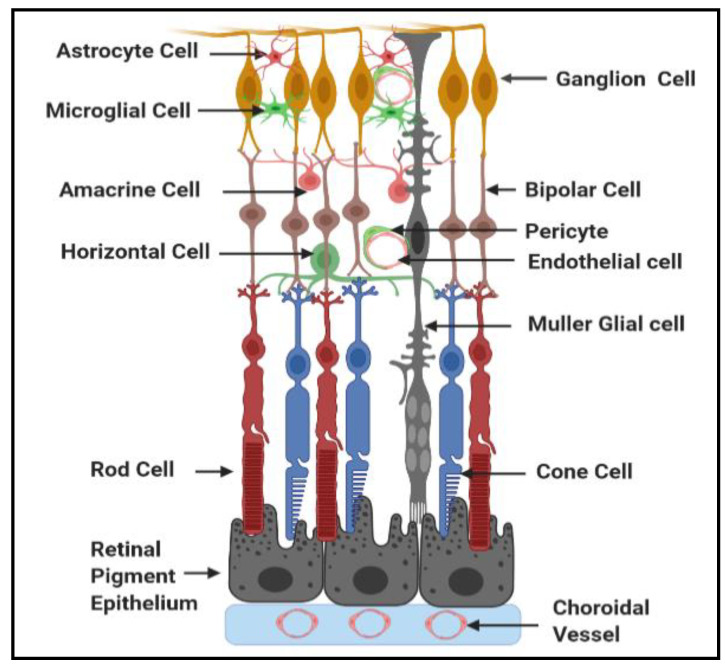
Schematic diagram depicting the interaction among different cell types in the retina.

The retina is a neurovascular tissue which has several layers of neurons interconnected by synapses (Figure 1). Rod and cone photoreceptor cells represent the neurons of outer retina, while bipolar, horizontal, amacrine, and ganglion cells represent the neurons of the inner retina. Neural signals from photoreceptor cells are processed by the second order neurons, bipolar and horizontal cells. Then, signals from these second order neurons target the retinal ganglion cells (RGCs) of the inner retina [24]. The neurons of the outer and inner of retina integrate to perform sensory functions and define color perception, spatial resolution, and contrast discrimination [25].

Because retinal neurons require an exceptional amount of energy to execute visual function, a continuously supply of nutrients and oxygen is essential [26]. There are two distinct retinal vascular systems supplying nutrients and oxygen to the retinal neurons which are choriocapillaries and the central retinal artery. The choriocapillaris supplies nutrients and oxygen via diffusion to the avascular photoreceptor layer of the outer retina. For example, glucose fuel reaches the photoreceptors after travelling from the choriocapillaris through glucose transporters (GLUT 1 and GLUT3) present in a monolayer of pigmented cells situated between the neuroretina and the choroid called the retinal pigment epithelium (RPE). The RPE collaborates with the fenestrated choriocapillaries and Bruch’s membrane to form the outer blood retinal barrier (oBRB) [27,28,29].

On the other hand, the central retinal artery supplies nutrients and oxygen to the inner retinal neurons [26]. The endothelial cells line the lumen of the microvasculature, act as a physical barrier between blood and surrounding tissue, and play a critical role in regulating the retinal homeostasis [30]. Pericytes wrap around the retinal capillaries and modulate the endothelial cell function [24]. In addition to the maintenance of vessel wall structural support, pericyte coverage regulates the expression of tight junction proteins in the adjacent endothelial cells [24]. A basal membrane separates pericytes from endothelial cells; however, holes in this membrane matrix allow the formation of cell to cell junctions between the pericytes and endothelial cells [31,32]. Matea and Newman [33] were the first to popularize the term neurovascular unit in the inner retina to describe the interactions between neurons, Müller glial cells, and vascular cells in controlling blood flow. Müller glial cells and astrocytes are two types of glial cells present between the neurons and the retinal vasculature which provide nutritional and regulatory support of neurons [34]. Müller glial cells span the entire retina from the retinal pigment epithelium to the inner limiting membrane and participate in vascular responses to meet the metabolic demands of the neurons by interchanging metabolites including lactate and amino acids from the circulation [35]. Furthermore, Müller glial cells are actively involved in regulating the glutamate/glutamine cycle, which is critical to control neurotransmission to protect from glutamate excitotoxicity, and to regulate blood–retinal barrier properties [35]. Astrocytes synapse on blood vessels to maintain autoregulation [36]. Microglia are a heterogenous population of resident macrophages that interact with neurons, glia, and endothelium to monitor local cellular and synaptic activity [37]. Microglia are responsive to changes in retinal environment, and they can be triggered by proinflammatory cytokines, damaged cells, or any immune-stimulatory agents. Activated microglia have the ability to respond to stress by releasing proinflammatory cytokines [38] and dispose of the dying cells via phagocytosis [39]. However, if microglia are sustained in an activated state, the secreted cytokines can affect other cell types in the proximity, particularly neuronal and vascular cells, bringing about the progression of many retinal diseases, including DR [40]. In summary, retinal neuronal cells are protected from harmful molecules in the circulation by oBRB, comprised of RPE, and the inner BRB (iBRB), comprised of endothelial cells [41]. Tight junctions between neighboring RPE cells and endothelial cells play a regulatory role in the strict control of fluid and solutes crossing the blood–retinal barrier, and they prevent the entrance of toxic molecules and plasma components into the retina [41]. To perform normal visual functions, highly coordinated activity of neuronal, glial, microglial, and microvasculature is required.

### 1.2. Metabolic Pathways Implicated in DR

**Figure 2:**
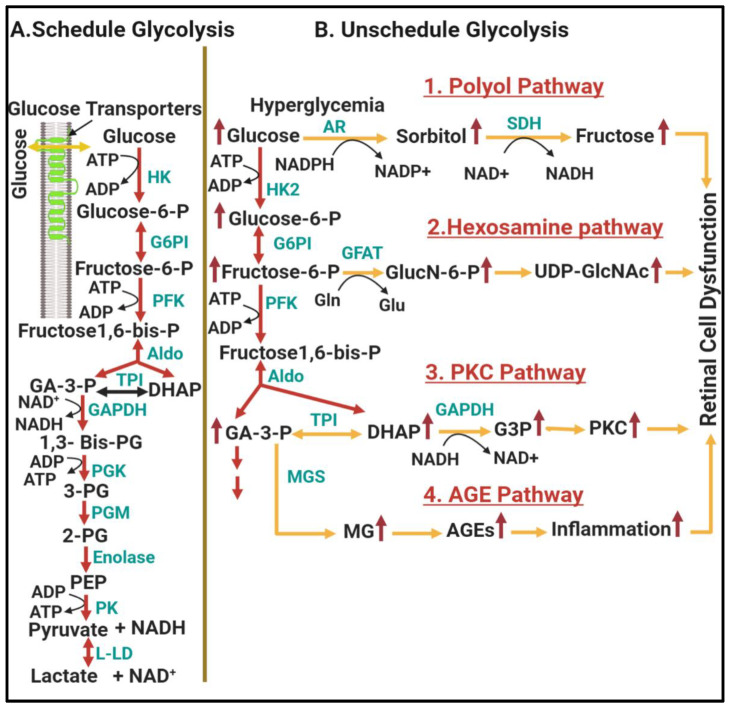
The contribution of unscheduled glycolysis to diabetic retinopathy. (**A**) represents key regulatory steps of scheduled glycolysis, while (**B**) represents hyperglycemia-linked glycolytic overload and metabolic dysfunction in unscheduled glycolysis resulting from activation of the polyol, hexosamine, PKC, and AGEs pathways, which further cause oxidative stress and inflammation leading to retinal cell dysfunction. Yellow arrows depict metabolic dysfunction in unscheduled glycolysis. Abbreviations: Glucose-6-P, glucose-6-phosphate; Fructose-6-P, fructose-6-phosphate; Fructose 1,6-bis-P, fructose 1,6-bis-Phosphate; GA-3-P, glyceraldehyde-3-phosphate; DHAP, dihydroxyacetone phosphate; 1,3 bis PG, 1,3 diphosphoglycerate; 3-PG, 3-phosphoglycerate; 2-PG; 2-phosphoglycerate; PEP, phosphoenolpyruvate; HK, hexokinase; G6PI, glucose 6-phosphate isomerase; PFK, phosphofructokinase; TPI, triose phosphate isomerase; Aldo, aldolase; GAPDH, glyceraldehyde-3-phosphate dehydrogenase; PGK, phosphoglycerate kinase; PGM, phosphoglycerate mutase; PK, pyruvate kinase; L-LD, L-lactic dehydrogenase; HK2, hexokinase2; OS, osmotic stress; AR, aldolase reductase; SDH, sorbitol dehydrogenase; GlucN-6-P, glucosamine-6-P; UDP-GlcNAc, uridine diphosphate-N-acetylhexosamine; GFAT, glutamine fructose-6-P amidotransferase; Gln, glutamine; Glu, glutamate; G3P, glycerol-3-P; PKC, protein kinase C; MG, methylglycoxal; MGS, methylglycoxal synthase AGE, advance glycation end-products. The figure is adopted with modification from [43].

The maintenance of glucose homeostasis is regulated by interplay between glycolysis, Krebs cycle, and OxPhos. Two intriguing features make glycolysis the most widespread metabolic pathway found in Earth’s life: (1) it does not require oxygen nor (2) compartmentalization by internal membranes, as the Krebs cycle and OxPhos do. These make glycolysis a more accessible source of ATP. In general, the glycolysis pathway primarily involves the intracellular breakdown of glucose into pyruvate or lactate following transporter-mediated glucose uptake. During this process, different intermediates are generated that are used as substrates for energy production as well as substrates for energy storage via the pathways of glycogenesis and lipogenesis [42]. Glycolysis is a nine-reaction pathway that is divided into two phases; upper and lower glycolysis. Upper glycolysis is an “investment phase” where ATP is consumed to set up the reactions of lower glycolysis, the “payoff phase” where ATP is generated. Upper glycolysis converts glucose into two trioses (dihydroxyacetone phosphate (DHAP) and glyceraldehyde 3-phosphate (GA-3P)) after conversion through three intermediates, glucose 6-phosphate (G6P), fructose 6-phosphate (F6P), and fructose 1,6-bisphosphate, generated by hexokinase (HK), phosphoglucose Isomerase (PGI), and phosphofructokinase (PFK), respectively. On the other hand, lower glycolysis converts the trioses into pyruvate or lactic acid through three intermediates, 3-phosphoglycerate (3-PG), 2-phosphoglycerate (2-PG), and phosphoenolpyyruvate (PEP), generated by phosphoglycerate kinase (PGK), phosphoglycerate mutase (PGM), enolase (ENO), respectively. The connection between upper and lower glycolysis is regulated by the enzyme called glyceraldehyde 3-phosphate dehydrogenase (GAPDH), which catalyzes the conversion of GA-3P to 1,3-bisphosphateglycerate (1,3 Bis-PG) and then to 3-PG. Under physiological conditions, all tissues, including those with the highest concentrations of glycolytic enzymes such as the brain and the retina, regulate glycolysis tightly to maintain a steady-state concentration of glycolytic intermediates; this regulation is called scheduled or regulated glycolysis (Figure 2A). Nevertheless, increased flux through glycolysis, which is commonly seen under ischemic conditions, results in glycolytic overload or unscheduled glycolysis (Figure 2B), which generates large amounts of intermediate metabolites that can be shunted into different damaging pathways [43]. In the following section, we describe how an abnormal increase in the steady-state concentrations of upper and lower glycolytic intermediates leads to multiple damaging pathways in DR. More detailed understanding of this metabolic alteration may identify new targets for improved therapy of DR.

#### 1.2.1. Involvement of Upper Glycolysis in Glycolytic Overload during DR

In regulated or scheduled glycolysis, upper glycolysis is regulated at three rate-limiting steps: (1) glucose uptake (which is regulated by glucose transporters (GLUT)) (2) glucose phosphorylation (which is catalyzed by different forms of hexokinase isozymes), (3) the generation of fructose1,6-biphosphate (which is catalyzed PFK). Recent research evidence revealed that increased hexokinase 2 (HK2) activity and increased G6P concentration are linked to the initiation of unregulated or unscheduled glycolysis, which leads to an abnormal increase in steady-state concentrations of upper glycolytic intermediates, including G6P, DHAP, and GA3P [43]. The accumulation of these upper glycolytic intermediates has been shown to be linked to the development of DR through shunting into four pathways: the polyol pathway, hexosamine pathway, diacylglycerol-dependent activation of the protein kinase C pathway, and Amadori/advanced glycation end products (AGEs) pathway as detailed below:

##### Shunting into Polyol Pathway

In regulated or scheduled glycolysis, glucose is normally phosphorylated by hexokinase, but when there is excess glucose uptake, hexokinase becomes saturated and any excess glucose is shunted into the polyol pathway. The polyol pathway is a two-step metabolic pathway in which glucose is reduced to sorbitol by a rate limiting enzyme known as aldose reductase using NADPH as a cofactor. Then, the enzyme sorbitol dehydrogenase converts the sorbitol to fructose using the cofactor NAD^+^ [44].

A hyperglycemia-induced increase in aldose reductase concentration is correlated with increased damage to certain cells of the retina, including endothelial cells [45,46,47], pericytes [48,49], ganglion cells [45], Müller glial cells [45], retinal pigment epithelial cells [45], and neurons [50]. The detrimental effects of the polyol pathway in DR could be explained by several mechanisms. First, activation of polyol pathway lowers the concentration of NADPH and NAD^+^, which are required for regeneration of glutathione, and consequently results in decreases in antioxidant defenses and overproduction of reactive oxygen species (ROS), leading to oxidative stress [51]. Second, as sorbitol is less permeable through the cell membranes, sorbitol accumulation within retinal cells during hyperglycemia causes cellular hyperosmolality, which subsequently induces an increase in intracellular water and osmotic damage [52]. Third, the fructose produced in the polyol pathway during diabetes is phosphorylated to fructose-3-phosphate, which in turn is broken down to 3-deoxyglucose and 3-deoxyglucosone. These two compounds play an important role in the production of AGEs [53,54]. Taken together, shunting glucose metabolism into the polyol pathway due to overwhelming upper glycolysis contributes to DR by inducing osmotic as well as oxidative stress and AGE formation in different retinal cells.

##### Shunting into Hexosamine Pathway

In unscheduled glycolysis, the accumulation of the upper glycolytic intermediate fructose-6-phosphate leads to the formation of N-acetyl glucosamine-6-phosphate (GlucN-6-P) by glutamine fructose-6-phosphate-amido transferase (GFAT), the rate limiting enzyme in the hexosamine pathway [55]. GlucN-6-P is rapidly transformed into uridine-5-diphosphate (UDP)-N-acetylglucosamine (UDP-GlcNAc), which is a precursor for all other amino sugars required for the biosynthesis of glycoproteins, glycolipids, proteoglycans, and glycosaminoglycans. While a small fraction of glucose is metabolized through hexosamine pathway under euglycemia, the hexosamine pathway is overstimulated under hyperglycemic condition, resulting in excess protein glycosylation that may disrupt gene expression and cellular functions in the retina, especially in neurovascular cells (reviewed in [56]). The contribution of shunting glucose metabolism into the hexosamine pathway to the pathogenesis of DR has been attributed to multiple mechanisms. Firstly, it increases retinal neuronal cell death by blocking the neuroprotective effect of the insulin/Akt signaling pathway [57]. Secondly, it promotes retinal ganglion cell death in the diabetic mouse model by increasing O-GlcNAcylation of NF-κB p65 subunit and its activation [58,59]. Thirdly, activation of hexosamine pathway increases O-GlcNAcylation of p53, which is associated with increased retinal pericyte apoptosis, leading to the early DR vascular dysfunction [58,60]. Finally, recent research reported that under hyperglycemic conditions, the product of the hexosamine pathway, UDP-N-acetyl glucosamine, competes with phosphorylation at post-translation modification sites on transcription factors, which disrupts the phosphorylation-mediated regulation of inflammatory responses that normally occurs through transcription growth factor-β [61]. In summary, the hexosamine pathway is activated when there is an excess of upper glycolytic intermediate fructose-6-phosphate that cannot be drained by glycolysis, and the activation of this pathway leads to DR, mainly through stabilizing transcription factor–promoter interactions that are well-known to be involved in DR.

##### Shunting into Diacylglycerol (Dag)/Protein Kinase C (PKC) Pathway

Under hyperglycemic conditions, the high intracellular glucose concentration causes accumulation of the upper glycolytic intermediate GA-3P, which then further enhances the production of DAG. DAG activates different isoforms of PKC within the cell [62]. Activation of PKC has been linked to the pathogenesis of DR at several levels. First, activation of PKC under hyperglycemic conditions causes retinal vascular dysfunction and pericyte losses [62,63,64]. Second, hyperglycemia-induced accumulation of DAG activates PKC-β, which alters the enzyme activities of NO, ET-1 and VEGF in endothelial cells and leads to vascular dysfunction. Third, hyperglycemia-induced accumulation of DAG also activates PKC-δ, which induces pericyte loss by two distinct pathways: (1) increasing ROS production and NF-κB activation and (2) upregulating the expression of a protein tyrosine phosphatase, SHP-1, to weaken the important survival signaling pathway of platelet-derived growth factor (PDGF) [62]. Thus, in unscheduled glycolysis, the accumulation of upper glycolysis trioses leads to DAG formation and downstream activation of several PKC isoforms, which are likely to be responsible for metabolic dysfunction in DR.

##### Shunting into Glycation End Products (Amadori/AGEs) Pathway

Glycation end products play an important role in the pathogenesis of DR [65,66,67,68]. Intracellular production of glycation end products involves nonenzymatic-glycation reactions between reducing sugars in upper glycolic metabolites and the free amino groups in proteins, lipids, and DNA. The formation of glycation end products begins with the formation of an unstable Schiff base between active aldehyde in reducing sugar and an amino group, which spontaneously rearranges itself into a relatively stable Amadori adduct [65,66]. Studies from our group recognized Amadori-glycated albumin (AGA)/inflammation cascade as a mechanism contributing to DR via activating microglia to secrete their inflammatory cytokines [67]. Further molecular rearrangements of AGA lead to the generation of AGEs [65]. In addition, methylglyoxal, which is formed from upper glycolysis metabolite, DHAP, by the enzyme methylglyoxal synthase, reacts with amino acids in proteins to generate AGEs. AGEs can interact with a wide range of cell surface AGE-binding receptors, including receptors for AGEs (RAGEs), leading to the formation of prooxidants and the activation of proinflammatory molecules [69]. RAGE is a member of an immunoglobulin receptors superfamily that is ubiquitously expressed in various retinal cells and is reported to be upregulated in diabetic patients. Binding of AGEs to RAGEs induces the activation of various downstream pathways, such as extracellular signal-regulated kinase 1/2, mitogen-activated protein kinases, and P38, which in turn activates NF-κB to induce the production of pro-inflammatory cytokines and/or oxidative stress [70,71]. AGEs affect cells by three main mechanisms: as adducts occurring on modified serum proteins, as endogenous adducts formed through glucose metabolism, or as extracellular matrix-immobilized modifications of long-lived structural proteins [72]. In DR, the accumulation of AGEs under hyperglycemic conditions evokes intracellular signaling cascades that lead to pericyte apoptosis and breakdown of the inner blood retinal barrier [73]. Moreover, hyperglycemia-induced AGE accumulation causes prolonged upregulation of the unfolded protein response (UPR) and autophagy via ER stress, which also promotes pericyte apoptosis [74]. Collectively, unscheduled glycolysis in diabetes accelerates the formation of AGEs, which act as major pathogenic mediators with several mechanism of actions leading to retinal cell damage.

#### 1.2.2. Involvement of Lower Glycolysis Overload in DR

**Figure 3:**
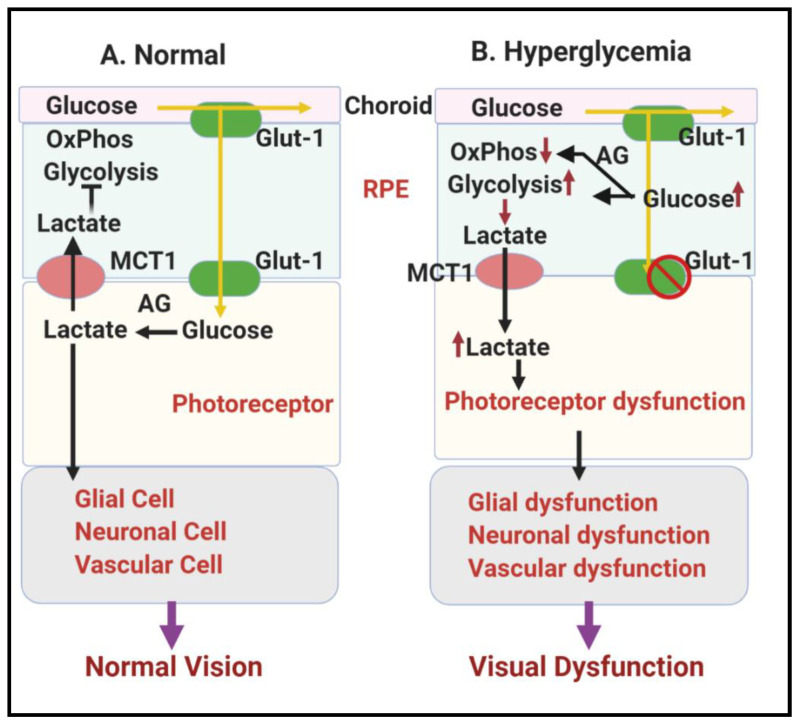
Schematic representation showing glucose metabolism in RPE and photoreceptors in the RPE-retinal ecosystem. (**A**) Normal glucose metabolism in RPE and photoreceptors in the RPE-retinal ecosystem under normal physiology leading to normal vision. (**B**) Hyperglycemia linked metabolic dysfunctions in RPE and photoreceptors in RPE-retinal ecosystem causing visual dysfunction in DR. Abbreviations: RPE, retinal pigment epithelium; Glut-1, Glucose transporter 1; MCT1, monocarboxylate transporter 1; AG, aerobic glycolysis.

In regulated or scheduled glycolysis, lower glycolysis transforms GA-3P into pyruvate or lactate in a series of enzymatic reactions. Contrary to upper glycolysis, these reactions are required to pay off ATPs invested in upper glycolysis, and therefore the generated intermediates cannot be shunted into other pathways. Lower glycolysis is mainly regulated at import and export of lactate (controlled by monocarboxylate transporter (MCT)). This highlights the importance of the final enzymes involved in pyruvate or lactate production, which are pyruvate kinase (PK) and lactate dehydrogenase A (LDHA), respectively, to glycolytic flux control. Rod and cone photoreceptor cells are the most metabolically active neurons in the outer retina [75]. Glucose that fuels photoreceptors comes from the choroidal blood and travels to the retina through a monolayer of RPE [29]. If the metabolic enzymes within the RPE do not consume the glucose, it moves down a concentration gradient toward the opposite side of the RPE cell, where it exits into the retina through transporters (Glut1 and 3) on the apical surface of the RPE [29]. Under normal conditions, photoreceptors, like tumor cells, mostly rely on aerobic glycolysis for their energy demand and release a substantial amount of lactate, even when oxygen is available [76,77,78]. Moreover, recent studies suggested that a balanced retinal metabolic ecosystem between RPEs, photoreceptors, and neuroglia is required to maintain proper function of each cell type [29,79]. Large amounts of lactate produced from photoreceptors are used as an alternative fuel in RPE cells and serve as a signal that prevents the RPE from utilizing glucose as an energy source, making more glucose available to the photoreceptors, as depicted in Figure 3A. However, under diabetic conditions, hyperglycemia results in metabolic reprogramming in RPE due to mitochondrial dysfunction [80,81], causing a glycolytic shift and lactate overproduction from RPE cells towards photoreceptors, which leads to photoreceptor cell death [29,79], as depicted in Figure 3B.

**Figure 4:**
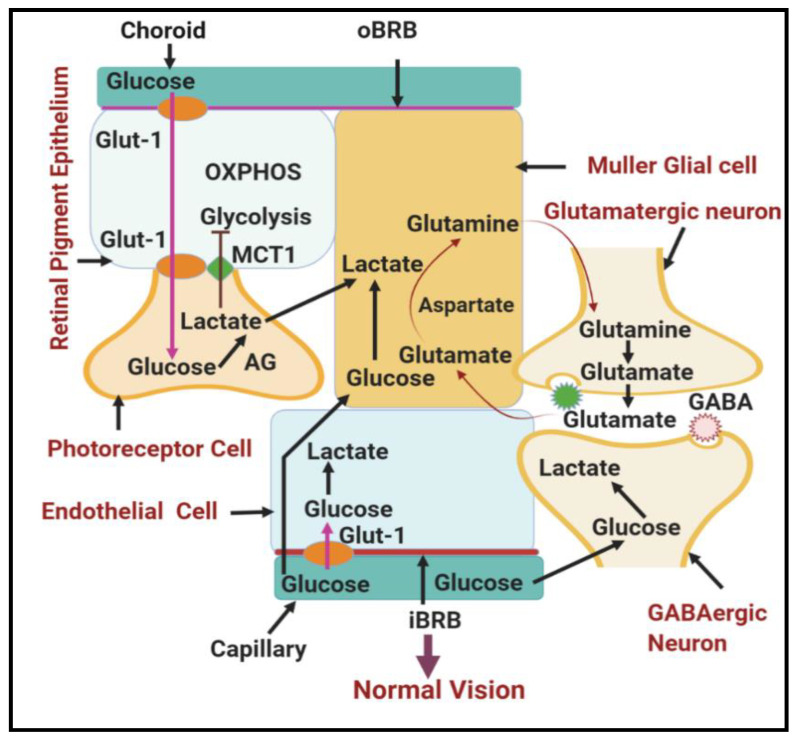
Schematic diagram showing normal glucose metabolism in different cells of the neurovascular unit in the retina. Different cell types in the neurovascular unit of the in retina form a metabolic ecosystem to perform normal vision. Abbreviations: RPE, retinal pigmented epithelium; Glut-1, glucose transporter 1; AG, aerobic glycolysis; OxPhos, oxidative phosphorylation; iBRB, inner blood–retinal barrier; oBRB, outer blood–retinal barrier; MCT1, monocarboxylate transporter.

**Figure 5:**
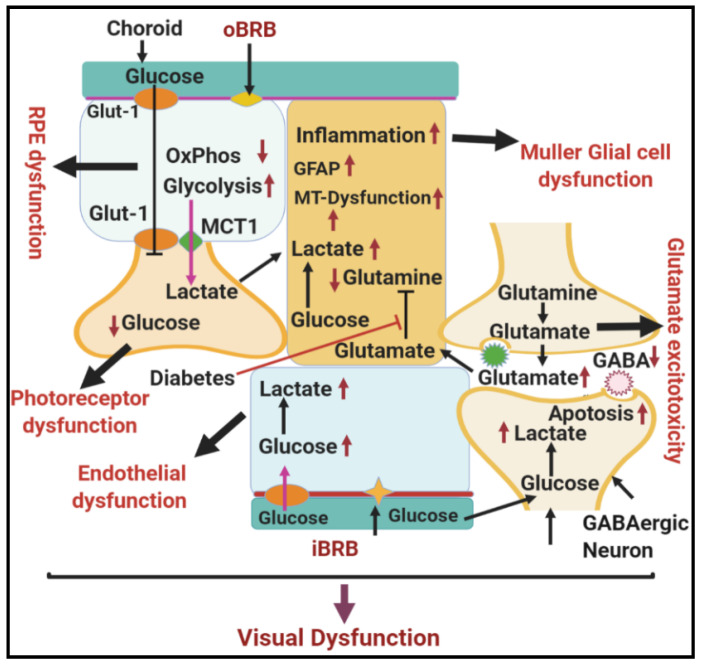
Schematic diagram showing the effect of hyperglycemia on metabolic dysfunction in the diabetic retina. Hyperglycemia-induced dysfunction of the different cell types in the neurovascular unit of the retina disrupts the entire metabolic ecosystem, leading to visual dysfunction in DR. Abbreviations: RPE, retinal pigmented epithelium; Glut-1, glucose transporter 1; AG, aerobic glycolysis; OxPhos, oxidative phosphorylation; iBRB, inner blood–retinal barrier; oBRB, outer blood–retinal barrier; MCT1, monocarboxylate transporter; GFAP, glial fibrillary acidic protein; GABA, gamma-aminobutyric acid.

In addition to RPE, photoreceptors export the lactate as fuel for neighboring Müller glial cells, which span the entire retina to provide structural and nutritional support to the retina [82]. In normal conditions, Müller glial cells convert lactate and aspartate to glutamine via the tricarboxylic acid cycle [1]. Glutamine produced by Müller glial cells is essential to drain the bulk of extracellular glutamate produced in the inner retina and to clear up glutamate around photoreceptor terminals, thus preventing glutamate neurotoxicity (Figure 4). However, during hyperglycemia, Müller glial cells seem to be particularly vulnerable to damage and are now recognized as potential players in the progression of DR [83,84]. For example, Müller glial cells in DR overexpress glial fibrillary acidic protein (GFAP) [84], showing decreased glutamine synthesis with subsequent accumulation of lactate and glutamate within Müller glial cells and around neurons, with resultant retinal excitotoxicity and photoreceptor damage [85] (Figure 5). Müller glial cells are also preferably positioned to mediate the neurovascular coupling in the retina as they are interposed between the retinal vasculature and the neurons and often enveloped in the vasculature [86]; thus, DR-induced Müller cell dysfunction further contributes to reduced interaction between the neurons and retinal vasculature.

In a healthy state, most endothelial cells remain quiescent and take part in maintaining barrier function and tissue homeostasis [87]. However, hyperglycemia induces oxidative stress in retinal endothelial cells (RECs), which further activates and perturbs several metabolic pathways, resulting in a self-perpetuating cycle of detrimental oxidative stress that advances the development of DR [88,89]. Hyperglycemia-induced oxidative stress in RECs originates from initiating mitochondrial dysfunction [90]. The overproduction of mitochondrial ROS under hyperglycemic conditions induces mitochondrial fragmentation and dysfunction, shifting the REC metabolism to hyperglycolysis, and reduces the ability of RECs to maintain both barrier function and tissue homeostasis in DR [91,92].

### 1.3. Other Biochemical and Molecular Changes Related to Dysregulation of Glycolysis in DR

In addition to the aforementioned involvement of glycolytic overload in DR, disrupting the balance between glycolysis and OxPhos leads to other biochemical and molecular changes seen in DR, such as synergistic crosstalk between hypoxia and hyperglycemia, endoplasmic reticulum (ER)-mitochondria miscommunication, and mitophagy dysregulation. Each of these will be briefly described in the following section.

#### 1.3.1. Synergistic Relationship between Hypoxia and Hyperglycemia in DR

Previous studies have reported that retinal tissue hypoxia occurs in DR of zebrafish, rodents, cats, dogs, and primates [93,94,95,96,97]. High glucose induces increased retinal vessel permeability, leakage of harmful substances, and degeneration of capillaries in early-stage DR, resulting in retinal hypoxia in the late stage of DR [98,99]. Retinal tissue hypoxia is a key mediator in the pathogenesis of DR [100]. A recent study showed that hyperglycemia induces cellular hypoxia through the production of mitochondrial ROS followed by the suppression of aquaporin’s (AQP) water transport function [101]. Among different APQs present in the retina, APQ4 is found mainly in Müller cell processes in reverse polarity, where they are in contact with retinal capillaries; therefore, efflux of water into the blood from the retina and the vitreous is reduced or prevented. Thus, dysregulation of aquaporin expression and orientation under hyperglycemia and hypoxia leads to Müller cell swelling, retinal edema and inflammation in DR. The cellular response to hypoxia is mediated via the activation of hypoxia-inducible factors (HIFs), consisting of oxygen sensing HIFα subunits and HIFβ [101]. Under normoxic conditions, transcriptional activity of HIF is inactivated, as the HIF1α/HIFβ complex is dissociated. However, during hypoxia, HIF1α dimerizes with HIFβ and then translocates to the nucleus, leading to downstream activation of a number of different pro-inflammatory cytokines (IL-1β, TNF-α, ICAM-1) [102,103,104] and many proangiogenic factors, such as fibroblast growth factors (FGFs), vascular endothelial growth factors (VEGFs), and platelet-derived growth factor (PDGF) [105,106,107]. In a normoxic state, there is a balance of angiogenic factors and endogenous anti-angiogenic factors. However, in a hypoxic state, there is breakdown in this balance of angiogenic factors and endogenous anti-angiogenic factors, leading to the neovascularization found in proliferative DR [108].

A recent study showed that hyperglycemia and hypoxia interact and have additive effects on the onset and progression of DR [109]. Retinal capillaries respond differently to acute and chronic hypoxia. Retinal vascular endothelial cells rapidly respond to acute hypoxia with the release of inflammatory cytokines [110], which recruits leukocytes and promotes their adherence and activation [111], causing obstruction at the retinal capillaries that leads to further hypoxia in the retina [64]. However, chronic hypoxia in the retina primarily induces expression of angiogenic growth factors [112], inducing retinal neovascularization in the retina of DR. Retinal neovascularization occurs adjacent to the nonperfused areas, supporting the hypothesis that hypoxic tissue releases angiogenic factors [64]. A plethora of angiogenic growth factors involved in retinal neovascularization are activated in a hypoxic state, but vascular endothelial growth factor (VEGF) has received considerable attention of late due to its potential as a therapeutic target [64]. VEGF is a potent angiogenic factor that stimulates endothelial cells to degrade the extracellular matrix, migrate, proliferate, and form tubes [113,114,115], and VEGF then acts as a survival factor for newly formed vessels [116]. VEGF interacts with cellular receptor tyrosine kinases, Flt-1 (VEGFR-1) and Flk-1/KDR (VEGFR-2), causing a cascade of events that stimulates angiogenic activities in endothelial cells [117]. Prior studies have reported an increased level of VEGF in the vitreous humor and retina of diabetic patients [118]). This increase is likely related to hypoxic VEGF induction, as increased levels of VEGF mRNA and protein are also seen in the retina of diabetic animals and human pathology specimens, especially in hypoxic regions near areas of neovascularization. [119,120]. A study in vitro also showed increased expression of VEGF mRNA in hypoxic retinal cells [121]. Under severe hypoxia, the expression of both hypoxic (HIF1α) and angiogenic factors (VEGF) is elevated in rodent retinal tissue [122]. Moreover, apoptosis occurs in retinal cells, including retinal RGCs and Müller glial cells, following severe hypoxia due to mitochondrial damage, which leads to an increased release of cytochrome c from the mitochondrial pore to the cytosol and subsequent activation of cytosolic caspase-3, resulting in the induction of apoptosis [123,124]. VEGF is the main factor causing retinal neovascularization and breakdown of the blood–retinal barrier (BRB), both of which contribute significantly to the DR disease process [125,126]. Targeted knock down of VEGF receptor 2 (VEGFR_2_), or its downstream target STAT3, can inhibit VEGF-induced neovascularization in the retinal endothelial cells of rats [127]. Hypoxia also upregulates glycolysis by increasing glucose transport activity through retinal capillary endothelial cells via increased GLUT1 expression, which is partially mediated by adenosine, A2R, and the cAMP-PKA pathway [128]. Knockdown of GLUT1 by siRNA inhibits GLUT1 expression and restricts glucose transport, thus decreasing retinal glucose concentrations and ameliorating the pathogenesis of DR in the retina of mice [129].

#### 1.3.2. Endoplasmic Reticulum-Mitochondria Miscommunication in Diabetic Retinopathy

The endoplasmic reticulum (ER) is a double membrane-bound organelle primarily involved in protein and lipid biosynthesis, protein folding and trafficking, and calcium homeostasis [130]. It is also involved in sensing metabolic changes within the cell and relaying signals of these changes to the nucleus for gene regulation [131]. This novel role of the ER is mediated by three major signal transducers: PKR-like endoplasmic reticulum kinase (PERK), inositol-requiring enzyme 1 (IRE1), and activating transcription factor 6 (ATF6) [130]. These proteins are activated in response either to increased accumulation of unfolded or misfolded proteins or to an upset in the Ca^2+^ ion homeostasis within the ER lumen, a condition known as ER stress [130]. The cells combat ER stress by activating IRE1, PERK and ATF6 signaling pathways, collectively known as the unfolded protein response [132,133], (erUPR), in which global protein synthesis is inhibited while expression of ER-targeted chaperones is enhanced. Unresolved ER stress activates pathological signaling pathways of oxidative stress and inflammation, resulting to retinal inflammation, apoptosis, and angiogenesis [75,134,135,136,137]. Recent studies have also demonstrated that dysfunction of ER is involved in the pathogenesis of DR [138,139].

Mitochondria are intracellular organelles involved in certain essential functions of the cell, including nutrient metabolism, ATP production, reactive oxygen production, intracellular Ca^2+^ regulation, cell survival, and cell death [140,141]. Their principal function is to synthesize ATP via OxPhos in concurrence with the oxidation of metabolites by Krebs’s cycle and β-oxidation of fatty acids [141]. Chronic hyperglycemia is linked with impaired mitochondrial functions, such as reduced OxPhos and subsequent reduced ATP production, overproduction of glycolytic intermediates, and subsequent reduction antioxidants, and overproduction of ROS [142,143,144]. The overproduction of ROS is associated with oxidative damage inflicted on lipids, DNA, and proteins [145]. Recent findings have established how ROS, pro-inflammatory cytokines (e.g., TNF-α), altered mitochondrial biogenesis, mtDNA damage, mitochondrial structural changes, or mitochondrial morphological changes all play a role in the pathogenesis of DR [142,143,144,146,147]. Moreover, a growing body of work reports the involvement of epigenetic modifications, alterations in methylation patterns of both mitochondrial and nuclear DNA and of histones, in the pathogenesis of DR [143,148].

Recent studies have proven that ER stress and mitochondrial dysfunction are significantly involved in the vascular and neuronal damage in DR [149]. The ER and mitochondria communicate directly via mitochondria associated ER membrane (MAM) [150], which permits reciprocal regulation of both organelles to allow coordinated regulation of various cellular activities, including energy metabolism, Ca^2+^ homeostasis [151], lipid homeostasis, cell survival, and cell death [152,153,154]. A previous study suggested that MAM dysfunction results in ROS overproduction and ATP underproduction, which further exacerbates ER stress and eventually leads to apoptotic cell death [155]. Furthermore, hyperglycemia reduces ER-mitochondria contact in retinal endothelial cells and perturbs MAM, which further progresses the development of DR [156].

#### 1.3.3. Mitophagy Dysregulation in Diabetic Retinopathy

As discussed above, mitochondria are the site of energy (ATP) production in cells via the electron transport chain (ETC) in the inner mitochondrial membrane. However, electrons also leak out of the ETC during ATP production, which are captured by molecular oxygen to generate highly reactive oxygen radicals/species (ROS), which then damage mitochondrial membrane lipids, proteins and mtDNA [144,157,158]. Although there are anti-oxidant systems in mitochondria to neutralize the ROS, including manganese superoxide dismutase (MnSOD), glutathione (GSH), and thioredoxin 2 (Trx2), these systems are overwhelmed by substantial amounts of ROS generated under sustained excess glucose in diabetes and DR [146,159]. The damaged or depolarized mitochondria are ineffective in producing ATP while they generate ROS. Therefore, removal of the damaged/dysfunctional mitochondria by lysosomal degradation via a specific autophagic process called mitophagy is critical [142,160,161]. For this, the damaged part of the mitochondrion is separated from the intact mitochondrion by fission involving dynamin-related protein 1 (DRP1) and fission protein fis1 [142]. The damaged mitochondria are then marked by ubiquitin via the PINK1-PARKIN pathway [162,163]. PINK1 is a kinase in the inner membrane of mitochondria; however, when the mitochondria are damaged/depolarized, PINK1 accumulates in the outer membrane and phosphorylates mitochondrial membrane proteins including the voltage dependent anion channel 1 (VDAC1) and mitofusin 2 (Mfn2) [164]. Thereafter, PARKIN, an E3 ubiquitin ligase, ubiquitinates mitochondrial membrane proteins to designate them for engulfment by a double membrane autophagophore containing ATG8 or microtubule-associated protein 1 light chain 3B (MAP1LC3B or LC3B). Mitochondrial adaptors such as optineurin and p62/sequestosome 1 recognize the ubiquitinated damaged mitochondria and LC3B for forming a double membrane autophagosome, within which the damaged mitochondria are entrapped. Subsequently, the autophagosome fuses with lysosomes to degrade the cargo and recycle it as nutrients. In addition, to maintain the optimal number of mitochondria and bioenergetics within a cell, the synthesis of new mitochondria (mitogenesis) and their fusion with existing mitochondria is critically important [165].

Mitogenesis involves transcription activation of nuclear DNA encoding mitochondria-targeted genes. A mitochondrion itself produces 13 of the ETC genes, while more than 1300 genes come from the nucleus [166]. The transcription coactivator peroxisome proliferator-activated receptor gamma coactivator 1 alpha (PGC1α) is an important factor for nuclear gene expression related to mitochondrial biogenesis, while transcription factor A, mitochondrial (TFAM) is involved in the synthesis of mitochondrial ETC genes. Therefore, a balance between mitophagy (removal of damaged mitochondria) and mitogenesis (synthesis of new mitochondria) is important. However, in DR, both mitophagy and mitogenesis are known to be dysregulated, and mitochondrial ATP production is reduced, leading to deficiencies in cellular biogenetics [142,157,160,167,168]. Similar to the energy demands of neurons of the central nervous system, the cells of the retina need a large amount of glucose and oxygen to generate ATP required for execution of visual function. On the other hand, under DR and mitochondrial dysfunction, ATP production slows down while ROS level increases. The accumulation of damaged mitochondria due to mitophagy defects results in the release of excess mtROS and oxidized mtDNA, which evoke an innate immune response involving NOD-like receptor family pyrin domain-containing 3 (NLRP3), caspase 1 activation, and release of active pro-inflammatory cytokines, interleukin IL-1α and IL-18 [158,160,161]. Therefore, under chronic hyperglycemia and DR, cellular redox stress and sterile inflammation play critical roles in the pathogenesis of DR [157,161].

We have further demonstrated that a protein called thioredoxin-interacting protein (TXNIP) is strongly induced by high glucose and DR in retinal cells [142,143,157,160,169,170]. TXNIP expression is maintained as long as hyperglycemia exists and causes cellular oxidative stress, inflammation and pre-mature cell death [171]. TXNIP’s actions include binding to the antioxidant and thiol reducing protein thioredoxin (Trx) and inhibition of its functions. Therefore, TXNIP causes cellular oxidative/nitrosative stress, organelle damage, and premature cell death under hyperglycemia and DR. We also published that TXNIP knockdown by siRNA in diabetic rat retinas prevents early molecular abnormalities of DR, which include retinal capillary basement membrane thickening, Müller cell activation (gliosis), and neuronal injury [169]. Furthermore, we have recently demonstrated that TXNIP upregulation and cellular redox stress cause mitochondrial dysfunction and mitophagic flux to lysosomes in retinal Müller glial cells and RPE [157,160,161]. However, excess mitophagic flux results in lysosomal enlargement and lysosomal membrane permeabilization, which releases lysosomal hydrolytic enzymes such as cathepsin B and L [160]. Cathepsin B may further act on mitochondrial membrane proteins and mediate mitochondrial damage and apoptosis [172]. TXNIP knockout by CRISPR and TXNIP gRNA or by siTXNIP prevents hyperglycemia-induced mitochondrial damage, ATP reduction, and excess mitophagic flux in Müller glial cells and RPE [142,160]. Therefore, we propose that TXNIP is a potential target for gene and drug therapy to prevent or slow down the progression of DR.

## 2. Conclusions

Chronic hyperglycemia affects the morphological and physiological function of different cell types of the eye, which ultimately leads to neurovascular dysfunction in DR. The incidence of DR continues to increase in both developing and developed countries as rates of obesity and diabetes also increase. Although there are some drugs which target VEGF for the treatment and management of DR, there is not yet an effective drug which can completely cure DR. The cellular and molecular mechanisms driving the pathogenesis of DR are very complex, and an interaction of many factors determines the onset of neurovascular dysfunction in DR. In recent years, metabolic dysfunction has been found to significantly contribute to the development of DR. Tightly regulated metabolic pathways are required to maintain a steady-state concentration of the various metabolic intermediates needed for normal physiological functioning of the eye. However, hyperglycemia disturbs the tight regulation of the different metabolic pathways, resulting in the intracellular accumulation of various metabolic intermediates. These intermediates further disrupt the metabolism and function of multiple cells types in neuro-vascular unit of the retina, eventually leading to DR. New ideas emerging from a metabolomics approach in the study of DR will drive the discovery of future therapeutic targets for the prevention and treatment of DR.

## References

[1] Harris Nwanyanwu K., Talwar N., Gardner T.W., Wrobel J.S., Herman W.H., Stein J.D. (2013). Predicting development of proliferative diabetic retinopathy. Diabetes Care.

[2] Klein R., Knudtson M.D., Lee K.E., Gangnon R., Klein B.E. (2009). The Wisconsin Epidemiologic Study of Diabetic Retinopathy XXIII: The twenty-five-year incidence of macular edema in persons with type 1 diabetes. Ophthalmology.

[3] Resnikoff S., Pascolini D., Etya’ale D., Kocur I., Pararajasegaram R., Pokharel G.P., Mariotti S.P. (2004). Global data on visual impairment in the year 2002. Bull. World Health Organ..

[4] Sivaprasad S., Gupta B., Gulliford M.C., Dodhia H., Mohamed M., Nagi D., Evans J.R. (2012). Ethnic variations in the prevalence of diabetic retinopathy in people with diabetes attending screening in the United Kingdom (DRIVE UK). PLoS ONE.

[5] Simó-Servat O., Hernández C., Simó R. (2013). Genetics in diabetic retinopathy: Current concepts and new insights. Curr. Genom..

[6] Yau J.W.Y., Rogers S.L., Kawasaki R., Lamoureux E.L., Kowalski J.W., Bek T., Chen S.-J., Dekker J.M., Fletcher A., Grauslund J. (2012). Global prevalence and major risk factors of diabetic retinopathy. Diabetes Care.

[7] Early Treatment Diabetic Retinopathy Study Research Group (2020). Grading Diabetic Retinopathy from Stereoscopic Color Fundus Photographs—An Extension of the Modified Airlie House Classification: ETDRS Report Number 10. Ophthalmology.

[8] The Diabetic Retinopathy Study Research Group (1976). Preliminary report on effects of photocoagulation therapy. Am. J. Ophthalmol..

[9] Fong D.S., Girach A., Boney A. (2007). Visual side effects of successful scatter laser photocoagulation surgery for proliferative diabetic retinopathy: A literature review. Retina.

[10] Wells J.A., Glassman A.R., Ayala A.R., Jampol L.M., Aiello L.P., Antoszyk A.N., Arnold-Bush B., Baker C.W., Bressler N.M., Diabetic Retinopathy Clinical Research Network (2015). Aflibercept, bevacizumab, or ranibizumab for diabetic macular edema. N. Engl. J. Med..

[11] Gross J.G., Glassman A.R., Jampol L.M., Inusah S., Aiello L.P., Antoszyk A.N., Baker C.W., Berger B.B., Bressler N.M., Writing Committee for the Diabetic Retinopathy Clinical Research Network (2015). Panretinal Photocoagulation vs Intravitreous Ranibizumab for Proliferative Diabetic Retinopathy: A Randomized Clinical Trial. JAMA.

[12] Kuiper E.J., Van Nieuwenhoven F.A., de Smet M.D., van Meurs J.C., Tanck M.W., Oliver N., Klaassen I., Van Noorden C.J., Goldschmeding R., Schlingemann R.O. (2008). The angio-fibrotic switch of VEGF and CTGF in proliferative diabetic retinopathy. PLoS ONE.

[13] Gross J.G., Glassman A.R., Liu D., Sun J.K., Antoszyk A.N., Baker C.W., Bressler N.M., Elman M.J., Ferris F.L., Gardner T.W. (2018). Five-Year Outcomes of Panretinal Photocoagulation vs Intravitreous Ranibizumab for Proliferative Diabetic Retinopathy: A Randomized Clinical Trial. JAMA Ophthalmol..

[14] Wolfe J.D., Shah A.R., Yonekawa Y., Al Faran A., Franklin M.S., Abbey A.M., Capone A. (2016). Receiver operating characteristic curve to predict anti-VEGF resistance in retinal vein occlusions and efficacy of Ozurdex. Eur. J. Ophthalmol..

[15] Krebs I., Glittenberg C., Ansari-Shahrezaei S., Hagen S., Steiner I., Binder S. (2013). Non-responders to treatment with antagonists of vascular endothelial growth factor in age-related macular degeneration. Br. J. Ophthalmol..

[16] Ehlken C., Jungmann S., Bohringer D., Agostini H.T., Junker B., Pielen A. (2014). Switch of anti-VEGF agents is an option for nonresponders in the treatment of AMD. Eye.

[17] Fogli S., Del Re M., Rofi E., Posarelli C., Figus M., Danesi R. (2018). Clinical pharmacology of intravitreal anti-VEGF drugs. Eye.

[18] Titchenell P.M., Antonetti D.A. (2013). Using the past to inform the future: Anti-VEGF therapy as a road map to develop novel therapies for diabetic retinopathy. Diabetes.

[19] Zehetner C., Bechrakis N.E., Stattin M., Kirchmair R., Ulmer H., Kralinger M.T., Kieselbach G.F. (2015). Systemic counterregulatory response of placental growth factor levels to intravitreal aflibercept therapy. Investig. Ophthalmol. Vis. Sci..

[20] Forbes J.M., Cooper M.E. (2013). Mechanisms of diabetic complications. Physiol. Rev..

[21] Chen Y., Zhao X., Wu H. (2019). Metabolic Stress and Cardiovascular Disease in Diabetes Mellitus: The Role of Protein O-GlcNAc Modification. Arterioscler. Thromb. Vasc. Biol..

[22] Gardner T.W., Davila J.R. (2017). The neurovascular unit and the pathophysiologic basis of diabetic retinopathy. Graefe’s Arch. Clin. Exp. Ophthalmol..

[23] Berg J.M., Tymoczko J.L., Stryer L. (2002). Section 16.1, Glycolysis Is an Energy-Conversion Pathway in Many Organisms. Biochemistry.

[24] Lechner J., O’Leary O.E., Stitt A.W. (2017). The pathology associated with diabetic retinopathy. Vis. Res..

[25] Masland R.H. (2001). The Fundamental Plan of the Retina Richard.

[26] Sun Y., Smith L.E.H. (2018). Retinal vasculature in development and diseases. Annu. Rev. Vis. Sci..

[27] Ban Y., Rizzolo L.J. (2000). Regulation of glucose transporters during development of the retinal pigment epithelium. Dev. Brain Res..

[28] Bergersen L., Jóhannsson E., Veruki M.L., Nagelhus E.A., Halestrap A., Sejersted O.M., Ottersen O.P. (1999). Cellular and subcellular expression of monocarboxylate transporters in the pigment epithelium and retina of the rat. Neuroscience.

[29] Kanow M.A., Giarmarco M.M., Jankowski C.S.R., Tsantilas K., Engel A.L., Du J., Linton J.D., Farnsworth C.C., Sloat S.R., Rountree A. (2017). Biochemical adaptations of the retina and retinal pigment epithelium support a metabolic ecosystem in the vertebrate eye. eLife.

[30] Chou J., Rollins S., Fawzi A.A. (2014). Role of endothelial cell and pericyte dysfunction in diabetic retinopathy: Review of techniques in rodent models. Adv. Exp. Med. Biol..

[31] Kur J., Newman E.A., Chan-Ling T. (2012). Cellular and physiological mechanisms underlying blood flow regulation in the retina and choroid in health and disease. Prog. Retin. Eye Res..

[32] Klaassen I., Van Noorden C.J., Schlingemann R.O. (2013). Molecular basis of the inner blood-retinal barrier and its breakdown in diabetic macular edema and other pathological conditions. Prog. Retin. Eye Res..

[33] Metea M.R., Newman E.A. (2007). Signalling within the neurovascular unit in the mammalian retina. Exp. Physiol..

[34] Antonetti D.A., Barber A.J., Bronson S.K., Freeman W.M., Gardner T.W., Jefferson L.S., Kester M., Kimball S.R., Krady J.K., LaNoue K.F. (2006). Diabetic retinopathy: Seeing beyond glucose-induced microvascular disease. Diabetes.

[35] Lieth E., LaNoue K.F., Antonetti D.A., Ratz M. (2000). Diabetes reduces glutamate oxidation and glutamine synthesis in the retina. Exp. Eye Res..

[36] Petzold G.C., Murthy V.N. (2011). Role of Astrocytes in Neurovascular Coupling. Neuron.

[37] Schafer D.P., Lehrman E.K., Kautzman A.G., Koyama R., Mardinly A.R., Yamasaki R., Ransohoff R.M., Greenberg M.E., Barres B.A., Stevens B. (2012). Microglia sculpt postnatal neural circuits in an activity and complement-dependent manner. Neuron.

[38] Krady J.K., Basu A., Allen C.M., Xu Y., LaNoue K.F., Gardner T.W., Levison S.W. (2005). Minocycline Reduces Proinflammatory Cytokine Expression, Microglial Activation, and Caspase-3 Activation in a Rodent Model of Diabetic Retinopathy. Diabetes.

[39] Elward K., Gasque P. (2003). “Eat me” and “don’t eat me” signals govern the innate immune response and tissue repair in the CNS: Emphasis on the critical role of the complement system. Mol. Immunol..

[40] Ibrahim A.S., El-Remessy A.B., Matragoon S., Zhang W., Patel Y., Khan S., Al-Gayyar M.M., El-Shishtawy M.M., Liou G.I. (2011). Retinal microglial activation and inflammation induced by amadori-glycated albumin in a rat model of diabetes. Diabetes.

[41] Simó R., Hernández C. (2014). Neurodegeneration in the diabetic eye: New insights and therapeutic perspectives. Trends Endocrinol. Metab..

[42] Rui L. (2014). Energy metabolism in the liver. Compr. Physiol..

[43] Rabbani N., Thornalley P.J. (2019). Hexokinase-2 Glycolytic Overload in Diabetes and Ischemia-Reperfusion Injury. Trends Endocrinol. Metab..

[44] Brownlee M. (2001). Biochemistry and molecular cell biology of diabetic complications. Nature.

[45] Chakrabarti S., Sima A.A.F., Nakajima T., Yagihashi S., Greene D.A. (1987). Aldose reductase in the BB rat: Isolation, immunological identification and localization in the retina and peripheral nerve. Diabetologia.

[46] Dagher Z., Park Y.S., Asnaghi V., Hoehn T., Gerhardinger C., Lorenzi M. (2004). Studies of rat and human retinas predict a role for the polyol pathway in human diabetic retinopathy. Diabetes.

[47] Drel V.R., Pacher P., Ali T.K., Shin J., Julius U., El-Remessy A.B., Obrosova I.G. (2008). Aldose reductase inhibitor fidarestat counteracts diabetes-associated cataract formation, retinal oxidative-nitrosative stress, glial activation, and apoptosis. Int. J. Mol. Med..

[48] Hohman T.C., Nishimura C., Robison W.G. (1989). Aldose reductase and polyol in cultured pericytes of human retinal capillaries. Exp. Eye Res..

[49] Li W., Chan L.S., Khatami M., Rockey J.H. (1986). Non-competitive inhibition of myo-inositol transport in cultured bovine retinal capillary pericytes by glucose and reversal by Sorbinil. BBA Biomembr..

[50] Cheung N., Mitchell P., Wong T.Y. (2010). Diabetic retinopathy. Lancet.

[51] Barnett P.A., Gonzalez R.G., Chylack L.T., Cheng H.M. (1986). The effect of oxidation on sorbitol pathway kinetics. Diabetes.

[52] Mathebula S.D. (2015). Polyol pathway: A possible mechanism of diabetes complications in the eye. Afr. Vis. Eye Health.

[53] Szwergold B.S., Kappler F., Brown T.R. (1990). Identification of fructose 3-phosphate in the lens of diabetic rats. Science.

[54] Yan L.-J. (2018). Redox imbalance stress in diabetes mellitus: Role of the polyol pathway. Anim. Models Exp. Med..

[55] Buse M.G. (2006). Hexosamines, insulin resistance, and the complications of diabetes: Current status. Am. J. Physiol. Endocrinol. Metab..

[56] Mathebula S.D. (2018). Biochemical changes in diabetic retinopathy triggered by hyperglycaemia: A review. Afr. Vis. Eye Health.

[57] Kim B.J., Silverman S.M., Liu Y., Wordinger R.J., Pang I.H., Clark A.F. (2016). In vitro and in vivo neuroprotective effects of cJun N-terminal kinase inhibitors on retinal ganglion cells. Mol. Neurodegener..

[58] Gurel Z., Sheibani N. (2018). O-Linked β-N-acetylglucosamine (O-GlcNAc) modification: A new pathway to decode pathogenesis of diabetic retinopathy. Clin. Sci..

[59] Watanabe T., Raff M.C. (1988). Retinal astrocytes are immigrants from the optic nerve. Nature.

[60] Gurel Z., Sieg K.M., Shallow K.D., Sorenson C.M., Sheibani N. (2013). Retinal O-linked N-acetylglucosamine protein modifications: Implications for postnatal retinal vascularization and the pathogenesis of diabetic retinopathy. Mol. Vis..

[61] Filla L.A., Edwards J.L. (2016). Metabolomics in diabetic complications. Mol. Biosyst..

[62] Geraldes P., King G.L. (2010). Activation of protein kinase C isoforms and its impact on diabetic complications. Circ. Res..

[63] Shin E.S., Sorenson C.M., Sheibani N. (2015). Diabetes and Retinal Vascular Dysfunction. J. Ophthalmic Vis. Res..

[64] Cai J., Boulton M. (2002). The pathogenesis of diabetic retinopathy: Old concepts and new questions. Eye.

[65] Tarr J.M., Kaul K., Chopra M., Kohner E.M., Chibber R. (2013). Pathophysiology of Diabetic Retinopathy. ISRN Ophthalmol..

[66] Stitt A.W. (2010). AGEs and diabetic retinopathy. Investig. Ophthalmol. Vis. Sci..

[67] Ibrahim A.S., El-Shishtawy M.M., Pena A., Liou G.I. (2010). Genistein attenuates retinal inflammation associated with diabetes by targeting of microglial activation. Mol. Vis..

[68] Ibrahim A.S., El-Shishtawy M.M., Zhang W., Caldwell R.B., Liou G.I. (2011). A((2)A) adenosine receptor (A((2)A)AR) as a therapeutic target in diabetic retinopathy. Am. J. Pathol..

[69] Hammes H.P., Brownlee M., Edelstein D., Saleck M., Martin S., Federlin K. (1994). Aminoguanidine inhibits the development of accelerated diabetic retinopathy in the spontaneous hypertensive rat. Diabetologia.

[70] Bierhaus A., Humpert P.M., Morcos M., Wendt T., Chavakis T., Arnold B., Stern D.M., Nawroth P.P. (2005). Understanding RAGE, the receptor for advanced glycation end products. J. Mol. Med..

[71] Milne R., Brownstein S. (2013). Advanced glycation end products and diabetic retinopathy. Amino Acids.

[72] Zong H., Ward M., Stitt A.W. (2011). AGEs, RAGE, and diabetic retinopathy. Curr. Diabetes Rep..

[73] Xu J., Chen L.J., Yu J., Wang H.J., Zhang F., Liu Q., Wu J. (2018). Involvement of Advanced Glycation End Products in the Pathogenesis of Diabetic Retinopathy. Cell. Physiol. Biochem..

[74] Fu D., Yu J.Y., Yang S., Wu M., Hammad S.M., Connell A.R., Du M., Chen J., Lyons T.J. (2016). Survival or death: A dual role for autophagy in stress-induced pericyte loss in diabetic retinopathy. Diabetologia.

[75] Du M., Wu M., Fu D., Yang S., Chen J., Wilson K., Lyons T.J. (2013). Effects of modified LDL and HDL on retinal pigment epithelial cells: A role in diabetic retinopathy?. Diabetologia.

[76] Du J., Rountree A., Cleghorn W.M., Contreras L., Lindsay K.J., Sadilek M., Gu H., Djukovic D., Raftery D., Satrústegui J. (2016). Phototransduction influences metabolic flux and nucleotide metabolism in mouse retina. J. Biol. Chem..

[77] Lindsay K.J., Du J., Sloat S.R., Contreras L., Linton J.D., Turner S.J., Sadilek M., Satrústegui J., Hurley J.B. (2014). Pyruvate kinase and aspartate-glutamate carrier distributions reveal key metabolic links between neurons and glia in retina. Proc. Natl. Acad. Sci. USA.

[78] Chinchore Y., Begaj T., Wu D., Drokhlyansky E., Cepko C.L. (2017). Glycolytic reliance promotes anabolism in photoreceptors. eLife.

[79] Brown E.E., DeWeerd A.J., Ildefonso C.J., Lewin A.S., Ash J.D. (2019). Mitochondrial oxidative stress in the retinal pigment epithelium (RPE) led to metabolic dysfunction in both the RPE and retinal photoreceptors. Redox Biol..

[80] Ola M.S., Berkich D.A., Xu Y., King M.T., Gardner T.W., Simpson I., LaNoue K.F. (2006). Analysis of glucose metabolism in diabetic rat retinas. Am. J. Physiol. Endocrinol. Metab..

[81] Sas K.M., Kayampilly P., Byun J., Nair V., Hinder L.M., Hur J., Zhang H., Lin C., Qi N.R., Michailidis G. (2016). Tissue-specific metabolic reprogramming drives nutrient flux in diabetic complications. JCI Insight.

[82] Kelly K., Wang J., Zhang S. (2018). The unfolded protein response signaling and retinal Müller cell metabolism. Neural Regen Res..

[83] Bringmann A., Pannicke T., Grosche J., Francke M., Wiedemann P., Skatchkov S.N., Osborne N.N., Reichenbach A. (2006). Muller cells in the healthy and diseased retina. Prog. Retin. Eye Res..

[84] Barber A.J., Lieth E., Khin S.A., Antonetti D.A., Buchanan A.G., Gardner T.W. (1998). Neural apoptosis in the retina during experimental and human diabetes. Early onset and effect of insulin. J. Clin. Investig..

[85] Li Q., Puro D.G. (2002). Diabetes-Induced Dysfunction of the Glutamate Transporter in Retinal Müller Cells. Investig. Ophthalmol. Vis. Sci..

[86] Newman E.A. (2013). Functional hyperemia and mechanisms of neurovascular coupling in the retinal vasculature. J. Cereb. Blood Flow Metab..

[87] De Zeeuw P., Wong B.W., Carmeliet P. (2015). Metabolic adaptations in diabetic endothelial cells. Circ. J..

[88] Kowluru R.A., Kanwar M., Kennedy A. (2007). Metabolic memory phenomenon and accumulation of peroxynitrite in retinal capillaries. Exp. Diabetes Res..

[89] Li C., Miao X., Li F., Wang S., Liu Q., Wang Y., Sun J. (2017). Oxidative Stress-Related Mechanisms and Antioxidant Therapy in Diabetic Retinopathy. Oxid. Med. Cell. Longev..

[90] Busik J.V., Mohr S., Grant M.B. (2008). Hyperglycemia-Induced reactive oxygen species toxicity to endothelial cells is dependent on paracrine mediators. Diabetes.

[91] Trudeau K., Molina A.J.A., Guo W., Roy S. (2010). High glucose disrupts mitochondrial morphology in retinal endothelial cells: Implications for diabetic retinopathy. Am. J. Pathol..

[92] Madsen-Bouterse S.A., Mohammad G., Kanwar M., Kowluru R.A. (2010). Role of mitochondrial DNA damage in the development of diabetic retinopathy, and the metabolic memory phenomenon associated with its progression. Antioxid. Redox Signal..

[93] Cao R., Jensen L.D.E., Söll I., Hauptmann G., Cao Y. (2008). Hypoxia-induced retinal angiogenesis in zebrafish as a model to study retinopathy. PLoS ONE.

[94] De Gooyer T.E., Stevenson K.A., Humphries P., Simpson D.A.C., Gardiner T.A., Stitt A.W. (2006). Retinopathy is reduced during experimental diabetes in a mouse model of outer retinal degeneration. Investig. Ophthalmol. Vis. Sci..

[95] Linsenmeier R.A., Braun R.D., McRipley M.A., Padnick L.B., Ahmed J., Hatchell D.L., McLeod D.S., Lutty G.A. (1998). Retinal hypoxia in long-term diabetic cats. Investig. Ophthalmol. Vis. Sci..

[96] Lai A.K.W., Lo A. (2013). Animal Models of Diabetic Retinopathy: Summary and Comparison. J. Diabetes Res..

[97] Grossniklaus H.E., Kang S.J., Berglin L. (2010). Animal models of choroidal and retinal neovascularization. Prog. Retin. Eye Res..

[98] Arden G. (2011). Hypoxia and Oxidative Stress in the Causation of Diabetic Retinopathy. Curr. Diabetes Rev..

[99] Chronopoulos A., Trudeau K., Roy S., Huang H., Vinores S.A., Roy S. (2011). High Glucose-induced Altered Basement Membrane Composition and Structure Increases Trans-endothelial Permeability: Implications for Diabetic Retinopathy. Curr. Eye Res..

[100] Ekberg N.R., Eliasson S., Li Y.W., Zheng X., Chatzidionysiou K., Falhammar H., Gu H.F., Catrina S.-B. (2019). Protective Effect of the HIF-1A Pro582Ser Polymorphism on Severe Diabetic Retinopathy. J. Diabetes Res..

[101] Sada K., Nishikawa T., Kukidome D., Yoshinaga T., Kajihara N., Sonoda K., Senokuchi T., Motoshima H., Matsumura T., Araki E. (2016). Hyperglycemia Induces Cellular Hypoxia through Production of Mitochondrial ROS Followed by Suppression of Aquaporin-1. PLoS ONE.

[102] Kaur C. (2008). Hypoxia-ischemia and retinal ganglion cell damage. Clin. Ophthalmol..

[103] Joussen A.M., Poulaki V., Le M.L., Koizumi K., Esser C., Janicki H., Schraermeyer U., Kociok N., Fauser S., Kirchhof B. (2004). A central role for inflammation in the pathogenesis of diabetic retinopathy. FASEB J..

[104] Zheng L., Gong B., Hatala D.A., Kern T.S. (2007). Retinal Ischemia and Reperfusion Causes Capillary Degeneration: Similarities to Diabetes. Investig. Opthalmol. Vis. Sci..

[105] Stone J., Itin A., Alon T., Pe’Er J., Gnessin H., Chan-Ling T., Keshet E. (1995). Development of retinal vasculature is mediated by hypoxia-induced vascular endothelial growth factor (VEGF) expression by neuroglia. J. Neurosci..

[106] Xin X., Rodrigues M., Umapathi M., Kashiwabuchi F., Ma T., Babapoor-Farrokhran S., Wang S., Hu J., Bhutto I., Welsbie D.S. (2013). Hypoxic retinal Müller cells promote vascular permeability by HIF-1–dependent up-regulation of angiopoietin-like 4. In Proceedings of the Proceedings of the National Academy of Sciences. Proc. Natl. Acad. Sci. USA.

[107] Krock B.L., Skuli N., Simon M.C. (2011). Hypoxia-Induced Angiogenesis: Good and Evil. Genes Cancer.

[108] Das A., Stroud S., Mehta A., Rangasamy S. (2015). New treatments for diabetic retinopathy. Diabetes Obes. Metab..

[109] Nyengaard J.R., Ido Y., Kilo C., Williamson J.R. (2004). Interactions Between Hyperglycemia and Hypoxia: Implications for Diabetic Retinopathy. Diabetes.

[110] Gries F.A. (1995). Alternative therapeutic principles in the prevention of microvascular and neuropathic complications. Diabetes Res. Clin. Pract..

[111] Michiels C., Arnould T., Remacle J. (2000). Endothelial cell responses to hypoxia: Initiation of a cascade of cellular interactions. Biochim. Biophys. Acta (BBA) Bioenerg..

[112] Tailor A., Granger D.N. (2000). Role of adhesion molecules in vascular regulation and damage. Curr. Hypertens. Rep..

[113] Levy A.P., Levy N.S., Loscalzo J., Calderone A., Takahashi N., Yeo K.-T., Koren G., Colucci W.S., Goldberg M.A. (1995). Regulation of Vascular Endothelial Growth Factor in Cardiac Myocytes. Circ. Res..

[114] Aiello L.P., Arrigg P.G., Shah S.T., Keyt B.A., Avery R.L., Jampel H.D., Pasquale L.R., Thieme H., King G.L., Iwamoto M.A. (1994). Vascular Endothelial Growth Factor in Ocular Fluid of Patients with Diabetic Retinopathy and Other Retinal Disorders. N. Engl. J. Med..

[115] Penn J.S., Madan A., Caldwell R., Bartoli M., Hartnett M. (2008). Vascular endothelial growth factor in eye disease. Prog. Retin. Eye Res..

[116] Ferrara N. (2009). Vascular Endothelial Growth Factor. Arter. Thromb. Vasc. Biol..

[117] Lee H.K., Chauhan S.K., Kay E., Dana R. (2011). Flt-1 regulates vascular endothelial cell migration via a protein tyrosine kinase-7–dependent pathway. Blood.

[118] De Vries C., Escobedo J.A., Ueno H., Houck K., Ferrara N., Williams L.T. (1992). The fms-like tyrosine kinase, a receptor for vascular endothelial growth factor. Science.

[119] Gupta N., Mansoor S., Sharma A., Sapkal A., Sheth J., Falatoonzadeh P., Kuppermann B., Kenney M.C. (2013). Diabetic Retinopathy and VEGF. Open Ophthalmol. J..

[120] Simpson D.A., Murphy G.M., Bhaduri T., Gardiner T.A., Archer D.B., Stitt A.W. (1999). Expression of the VEGF Gene Family during Retinal Vaso-Obliteration and Hypoxia. Biochem. Biophys. Res. Commun..

[121] Rodrigues M., Xin X., Jee K., Babapoor-Farrokhran S., Kashiwabuchi F., Ma T., Bhutto I., Hassan S.J., Daoud Y., Baranano D. (2013). VEGF Secreted by Hypoxic Muller Cells Induces MMP-2 Expression and Activity in Endothelial Cells to Promote Retinal Neovascularization in Proliferative Diabetic Retinopathy. Diabetes.

[122] Kaur C., Sivakumar V., Foulds W.S. (2006). Early Response of Neurons and Glial Cells to Hypoxia in the Retina. Investig. Opthalmol. Vis. Sci..

[123] Lam T.T., Abler A.S., Tso M.O.M. (1999). Apoptosis and caspases after ischemia-reperfusion injury in rat retina. Investig. Ophthalmol. Vis. Sci..

[124] Kaur C., Sivakumar V., Foulds W.S., Luu C.D., Ling E.-A. (2012). Hypoxia-Induced Activation ofN-methyl-D-aspartate Receptors Causes Retinal Ganglion Cell Death in the Neonatal Retina. J. Neuropathol. Exp. Neurol..

[125] Frank R.N. (2004). Diabetic Retinopathy. N. Engl. J. Med..

[126] Skondra D., Noda K., Almulki L., Tayyari F., Frimmel S., Nakazawa T., Kim I.K., Zandi S., Thomas K.L., Miller J.W. (2008). Characterization of Azurocidin as a Permeability Factor in the Retina: Involvement in VEGF-Induced and Early Diabetic Blood-Retinal Barrier Breakdown. Investig. Opthalmol. Vis. Sci..

[127] Simmons A.B., Bretz C.A., Wang H., Kunz E., Hajj K., Kennedy C., Yang Z., Suwanmanee T., Kafri T., Hartnett M.E. (2018). Gene therapy knockdown of VEGFR2 in retinal endothelial cells to treat retinopathy. Angiogenesis.

[128] Takagi H., King G.L., Aiello L.P. (1998). Hypoxia upregulates glucose transport activity through an adenosine-mediated increase of GLUT1 expression in retinal capillary endothelial cells. Diabetes.

[129] You Z.-P., Zhang Y.-L., Shi K., Shi L., Zhang Y.-Z., Zhou Y., Wang C.-Y. (2017). Suppression of diabetic retinopathy with GLUT1 siRNA. Sci. Rep..

[130] Zhang J., Li Y., Jiang S., Yu H., An W. (2014). Enhanced endoplasmic reticulum SERCA activity by overexpression of hepatic stimulator substance gene prevents hepatic cells from ER stress-induced apoptosis. Am. J. Physiol. Physiol..

[131] Ron D., Walter P. (2007). Signal integration in the endoplasmic reticulum unfolded protein response. Nat. Rev. Mol. Cell Biol..

[132] Rao R.V., Ellerby H.M., Bredesen D.E. (2004). Coupling endoplasmic reticulum stress to the cell death program. Cell Death Differ..

[133] Lindholm D., Wootz H., Korhonen L. (2006). ER stress and neurodegenerative diseases. Cell Death Differ..

[134] Li J., Wang J.J., Yu Q., Wang M., Zhang S.X. (2009). Endoplasmic reticulum stress is implicated in retinal inflammation and diabetic retinopathy. FEBS Lett..

[135] Tang L., Zhang Y., Jiang Y., Willard L., Ortiz E., Wark L., Medeiros D.M., Lin D. (2011). Dietary wolfberry ameliorates retinal structure abnormalities in db/db mice at the early stage of diabetes. Exp. Biol. Med..

[136] Zhong Y., Li J., Chen Y., Wang J.J., Ratan R., Zhang S.X. (2012). Activation of Endoplasmic Reticulum Stress by Hyperglycemia Is Essential for Müller Cell-Derived Inflammatory Cytokine Production in Diabetes. Diabetes.

[137] Chen Y., Wang J.J., Li J., Hosoya K.I., Ratan R., Townes T., Zhang S.X. (2012). Activating transcription factor 4 mediates hyperglycaemia-induced endothelial inflammation and retinal vascular leakage through activation of STAT3 in a mouse model of type 1 diabetes. Diabetologia.

[138] Elmasry K., Ibrahim A.S., Saleh H., Elsherbiny N., Elshafey S., Hussein K.A., Al-Shabrawey M. (2018). Role of endoplasmic reticulum stress in 12/15-lipoxygenase-induced retinal microvascular dysfunction in a mouse model of diabetic retinopathy. Diabetologia.

[139] Jing G., Wang J.J., Zhang S.X. (2011). ER Stress and Apoptosis: A New Mechanism for Retinal Cell Death. Exp. Diabetes Res..

[140] Kim J.-A., Wei Y., Sowers J.R. (2008). Role of Mitochondrial Dysfunction in Insulin Resistance. Circ. Res..

[141] Bhatti J.S., Bhatti G.K., Reddy P.H. (2017). Mitochondrial dysfunction and oxidative stress in metabolic disorders—A step towards mitochondria based therapeutic strategies. Biochim. Biophys. Acta (BBA) Mol. Basis Dis..

[142] Devi T.S., Somayajulu M., Kowluru R.A., Singh L.P. (2017). TXNIP regulates mitophagy in retinal Müller cells under high-glucose conditions: Implications for diabetic retinopathy. Cell Death Dis..

[143] Perrone L., Devi T.S., Hosoya K.-I., Terasaki T., Singh L.P. (2009). Thioredoxin interacting protein (TXNIP) induces inflammation through chromatin modification in retinal capillary endothelial cells under diabetic conditions. J. Cell. Physiol..

[144] Singh L.P. (2017). The Role of Txnip in Mitophagy Dysregulation and Inflammasome Activation in Diabetic Retinopathy: A New Perspective. JOJ Ophthalmol..

[145] Finkel T., Holbrook N.J. (2000). Oxidants, oxidative stress and the biology of ageing. Nat. Cell Biol..

[146] Kowluru R.A., Mishra M. (2015). Oxidative stress, mitochondrial damage and diabetic retinopathy. Biochim. Biophys. Acta (BBA) Mol. Basis Dis..

[147] Madsen-Bouterse S.A., Kowluru R.A. (2008). Oxidative stress and diabetic retinopathy: Pathophysiological mechanisms and treatment perspectives. Rev. Endocr. Metab. Disord..

[148] Kowluru R.A., Kowluru A., Mishra M., Kumar B. (2015). Oxidative stress and epigenetic modifications in the pathogenesis of diabetic retinopathy. Prog. Retin. Eye Res..

[149] Zhang S.X., Ma J.H., Bhatta M., Fliesler S.J., Wang J.J. (2015). The unfolded protein response in retinal vascular diseases: Implications and therapeutic potential beyond protein folding. Prog. Retin. Eye Res..

[150] López-Crisosto C., Bravo-Sagua R., Rodriguez-Peña M., Mera C., Castro P.F., Quest A.F.G., Rothermel B.A., Cifuentes M., Lavandero S. (2015). ER-to-mitochondria miscommunication and metabolic diseases. Biochim. Biophys. Acta (BBA) Mol. Basis Dis..

[151] Rizzuto R., Pinton P., Carrington W., Fay F.S., Fogarty K.E., Lifshitz L.M., Tuft R.A., Pozzan T. (1998). Close contacts with the endoplasmic reticulum as determinants of mitochondrial Ca^2+^ responses. Science.

[152] Bravo-Sagua R., Torrealba N., Paredes F., Morales P.E., Pennanen C., Lopez-Crisosto C., Troncoso R., Criollo A., Chiong M., Hill J.A. (2014). Organelle communication: Signaling crossroads between homeostasis and disease. Int. J. Biochem. Cell Biol..

[153] Marchi S., Patergnani S., Pinton P. (2014). The endoplasmic reticulum–mitochondria connection: One touch, multiple functions. Biochim. Biophys. Acta (BBA) Bioenerg..

[154] Hayashi T., Rizzuto R., Hajnoczky G., Su T.-P. (2009). MAM: More than just a housekeeper. Trends Cell Biol..

[155] Kornmann B. (2013). The molecular hug between the ER and the mitochondria. Curr. Opin. Cell Biol..

[156] Tubbs E., Theurey P., Vial G., Bendridi N., Bravard A., Chauvin M.-A., Ji-Cao J., Zoulim F., Bartosch B., Ovize M. (2014). Mitochondria-Associated Endoplasmic Reticulum Membrane (MAM) Integrity Is Required for Insulin Signaling and Is Implicated in Hepatic Insulin Resistance. Diabetes.

[157] Devi T.S., Lee I., Huttemann M., Kumar A., Nantwi K.D., Singh L.P. (2012). TXNIP links innate host defense mechanisms to oxidative stress and inflammation in retinal Muller glia under chronic hyperglycemia: Implications for diabetic retinopathy. Exp. Diabetes Res..

[158] Alfarhan M., Jafari E., Narayanan S.P. (2020). Acrolein: A Potential Mediator of Oxidative Damage in Diabetic Retinopathy. Biomolecules.

[159] Zhang L.W., Zhao H., Chen B.H. (2019). Reactive oxygen species mediates a metabolic memory of high glucose stress signaling in bovine retinal pericytes. Int. J. Ophthalmol..

[160] Devi T.S., Yumnamcha T., Yao F., Somayajulu M., Kowluru R.A., Singh L.P. (2019). TXNIP mediates high glucose-induced mitophagic flux and lysosome enlargement in human retinal pigment epithelial cells. Biol. Open.

[161] Yumnamcha T., Devi T.S., Singh L.P. (2019). Auranofin Mediates Mitochondrial Dysregulation and Inflammatory Cell Death in Human Retinal Pigment Epithelial Cells: Implications of Retinal Neurodegenerative Diseases. Front. Neurosci..

[162] Jin S.M., Youle R.J. (2012). PINK1- and Parkin-mediated mitophagy at a glance. J. Cell Sci..

[163] Killackey S.A., Philpott D.J., Girardin S.E. (2020). Mitophagy pathways in health and disease. J. Cell Biol..

[164] Bader V., Winklhofer K.F. (2020). Mitochondria at the interface between neurodegeneration and neuroinflammation. Semin. Cell Dev. Biol..

[165] Palikaras K., Lionaki E., Tavernarakis N. (2015). Coordination of mitophagy and mitochondrial biogenesis during ageing in C. elegans. Nature.

[166] Reynolds J.C., Bwiza C.P., Lee C. (2020). Mitonuclear genomics and aging. Hum. Genet..

[167] Santos J.M., Tewari S., Goldberg A.F., Kowluru R.A. (2011). Mitochondrial biogenesis and the development of diabetic retinopathy. Free Radic. Biol. Med..

[168] Hombrebueno J.R., Cairns L., Dutton L.R., Lyons T.J., Brazil D.P., Moynagh P., Curtis T.M., Xu H. (2019). Uncoupled turnover disrupts mitochondrial quality control in diabetic retinopathy. JCI Insight..

[169] Perrone L., Devi T.S., Hosoya K.I., Terasaki T., Singh L.P. (2010). Inhibition of TXNIP expression in vivo blocks early pathologies of diabetic retinopathy. Cell Death Dis.

[170] Devi T.S., Hosoya K.I., Terasaki T., Singh L.P. (2013). Critical role of TXNIP in oxidative stress, DNA damage and retinal pericyte apoptosis under high glucose: Implications for diabetic retinopathy. Exp. Cell Res..

[171] Singh L.P. (2013). Thioredoxin Interacting Protein (TXNIP) and Pathogenesis of Diabetic Retinopathy. J. Clin. Exp. Ophthalmol..

[172] Wang K., Zhan Y., Chen B., Lu Y., Yin T., Zhou S., Zhang W., Liu X., Du B., Wei X. (2020). Tubeimoside I-induced lung cancer cell death and the underlying crosstalk between lysosomes and mitochondria. Cell Death Dis.

